# Supplementary material for: Time-series transcriptome analysis identified differentially expressed genes in broiler chicken infected with mixed Eimeria species
Source: Front Genet. 2022 Aug 8;13:886781. doi: 10.3389/fgene.2022.886781 (PMC9393255; doi:10.3389/fgene.2022.886781)
Supplement: Supplementary file 2 [file DataSheet1.ZIP › 4dpi_KEGG.Gsea.1625100298584/KEGG_TERPENOID_BACKBONE_BIOSYNTHESIS.html]

Details for gene set KEGG\_TERPENOID\_BACKBONE\_BIOSYNTHESIS[GSEA]

|  || Dataset | TMM\_4dpi\_gct\_format\_4dpi\_gct\_format.Class\_4dpi.cls #PC\_versus\_NC.Class\_4dpi.cls #PC\_versus\_NC\_repos |
| Phenotype | Class\_4dpi.cls#PC\_versus\_NC\_repos |
| Upregulated in class | 1 |
| GeneSet | KEGG\_TERPENOID\_BACKBONE\_BIOSYNTHESIS |
| Enrichment Score (ES) | 0.7495593 |
| Normalized Enrichment Score (NES) | 1.8591883 |
| Nominal p-value | 0.0019004181 |
| FDR q-value | 0.033524383 |
| FWER p-Value | 0.0942 |
Table: GSEA Results Summary

  

Fig 1: Enrichment plot: KEGG\_TERPENOID\_BACKBONE\_BIOSYNTHESIS      
 Profile of the Running ES Score & Positions of GeneSet Members on the Rank Ordered List

  

| SYMBOL | TITLE | RANK IN GENE LIST | RANK METRIC SCORE | RUNNING ES | CORE ENRICHMENT || 1 | HMGCS1 | na | 30 | 2.183 | 0.2397 | Yes |
| 2 | IDI2 | na | 67 | 1.826 | 0.4392 | Yes |
| 3 | FDPS | na | 71 | 1.792 | 0.6377 | Yes |
| 4 | HMGCR | na | 299 | 1.179 | 0.7496 | Yes |
| 5 | MVD | na | 1777 | 0.461 | 0.6776 | No |
| 6 | PDSS1 | na | 1971 | 0.428 | 0.7089 | No |
| 7 | ACAT2 | na | 2179 | 0.392 | 0.7351 | No |
| 8 | MVK | na | 6801 | -0.071 | 0.3578 | No |
| 9 | PMVK | na | 7240 | -0.112 | 0.3338 | No |
| 10 | ACAT1 | na | 7276 | -0.116 | 0.3437 | No |
| 11 | GGPS1 | na | 7444 | -0.131 | 0.3443 | No |
| 12 | PDSS2 | na | 9331 | -0.326 | 0.2233 | No |
Table: GSEA details [plain text format]

  

Fig 2: KEGG\_TERPENOID\_BACKBONE\_BIOSYNTHESIS      
 Blue-Pink O' Gram in the Space of the Analyzed GeneSet

  

Fig 3: KEGG\_TERPENOID\_BACKBONE\_BIOSYNTHESIS: Random ES distribution      
 Gene set null distribution of ES for **KEGG\_TERPENOID\_BACKBONE\_BIOSYNTHESIS**
